# An Analysis of the Relationship between the Learning Process and Learning Motivation Profiles of Japanese Pharmacy Students Using Structural Equation Modeling

**DOI:** 10.3390/pharmacy6020035

**Published:** 2018-04-23

**Authors:** Shigeo Yamamura, Rieko Takehira

**Affiliations:** 1 Department of Biostatistics, Faculty of Pharmaceutical Sciences, Josai International University, Chiba 283-8555, Japan; 2 Department of Medical Psychology, Pharmaceutical Education Research Center, Kitasato University School of Pharmacy, Shirogane, Tokyo 108-8641, Japan; takehirar@pharm.kitasato-u.ac.jp

**Keywords:** learning motivation, learning process, pharmacy students, clinical clerkship, structural equation modeling, deep learning

## Abstract

Pharmacy students in Japan have to maintain strong motivation to learn for six years during their education. The authors explored the students’ learning structure. All pharmacy students in their 4th through to 6th year at Josai International University participated in the survey. The revised two factor study process questionnaire and science motivation questionnaire II were used to assess their learning process and learning motivation profiles, respectively. Structural equation modeling (SEM) was used to examine a causal relationship between the latent variables in the learning process and those in the learning motivation profile. The learning structure was modeled on the idea that the learning process affects the learning motivation profile of respondents. In the multi-group SEM, the estimated mean of the deep learning to learning motivation profile increased just after their clinical clerkship for 6th year students. This indicated that the clinical experience benefited students’ deep learning, which is probably because the experience of meeting with real patients encourages meaningful learning in pharmacy studies.

## 1. Introduction

In 2006, the duration of a four-year course in pharmacy education in Japan was extended to six years [1]. The new course aims to educate and enable pharmacists to provide patient-centered care. Along with extending the course term, practical pharmacy training programs were introduced: pre-practical training in their 4th year and practical training for 22 weeks in community pharmacies and hospital pharmacies in their 5th year as students. In their 6th year, most of the students return to pharmacy school and participate in a special research program [1]. As pharmacy education lasts 6 years, students must maintain strong motivation. In previous research, the authors noted a positive change in learning motivation based on self-determination and an inclination towards “deep learning” among students after the introduction of clinical clerkships [2,3]. The students were considered to be motivated during clinical clerkship and to understand the underlying concepts of pharmacy by deep learning [4]. 

The learning process and learning motivation profiles correlate with each other. To maintain the strength of students’ motivation for a long time, it is useful for faculty members to understand their learning structures in order to accommodate them [5]. Furthermore, learning motivation is an important factor for continued professional learning in the future [6]. Some reports on the learning process and learning motivation profiles of medical and nursing students have been published [7,8] and a few studies have also reported on pharmacy students [9]. 

However, a causal relationship between the learning motivation profile and learning process is usually unclear. If the causal relationship between learning motivation profiles and learning process can be established, effective curriculum to educate high motivated students in both pharmacy school and clinical sites can be developed. 

To educate a pharmacist as a life-long learner, clinical experience in practice training or clinical clerkship at clinical sites would be important. Early clinical experience could potentially influence future learning motivation. Therefore, it would be important to know how the practical training can influence their learning motivation. 

In previous papers, we modeled the learning process and learning motivation profiles of Japanese pharmacy students independently [2,3]. In this report, the authors established a model demonstrating the causal relationship between the learning process and learning motivation profiles using structural equation modeling (SEM). An effect of clinical clerkship on learning structure of pharmacy students was also investigated by multi-group analysis in SEM.

## 2. Materials and Methods 

### 2.1. Study Design: A Cross-Sectional Study Design Was Used

Sample: All 4th–6th year pharmacy students at Josai International University participated in the survey. A total of 165 completed responses (out of all 171 students) were used in the analysis (65 out of 69 4th-year students, 43 out of 45 5th-year students and 57 out of 57 6th-year students responded). Overall, the effective response rate was 93.2%. The survey was conducted at the beginning of the spring semester in early April 2016. After the students received a brief explanation of the survey’s purpose, they were given instructions on how to complete the questionnaire. If they were willing to participate, they signed a consent form, after which they answered the questionnaire. 

Questionnaire: The authors measured the students’ learning motivation profiles with the 25-item “science motivation questionnaire II (SMQ-II)” [10] (© 2011 Shawn M. Glynn). This instrument assesses the factors underlying the students’ motivation (intrinsic motivation: IM, career motivation: CM, self-determination: SD, self-efficacy and grade motivation: GM) to learn science [10]. The SMQ-II was also applied to evaluate their motivation in learning other subjects. A Japanese version of the SMQ-II was used after replacing *science* with *pharmacy* and making corrections for minor character errors [11]. The Likert data were ordinally scaled from 1 to 5 (1 = never, 2 = rarely, 3 = sometimes, 4 = usually, and 5 = always). The authors were given permission to use the questionnaire for this study. The average score and standard deviation for each item and each school year among the students were expressed in a previous paper [2]. The students’ learning process was measured with the 20-item “revised two factor study process questionnaire (R-SPQ-2F)” [12], which was translated into Japanese. The questionnaire was designed to serve as an empirical indicator of the deep approach (DA) and surface approach (SA) components of the students’ learning process. DA and SA have two indicators, respectively: DA has deep motive (DM) and deep strategy (DS), while SA has surface motive (SM) and surface strategy (SS). The students rated 20 items on a five-point scale: 1 means that the item is never or rarely true; 2 means that the item is sometimes true for me; 3 means that the item is true for me about half the time; 4 means that the item is frequently true for me; and 5 means the item is always or almost always true for me. John Biggs and David Kember own the copyright to the original R-SPQ-2F. They indicated that the questionnaire is allowed for research use upon fulfilling the conditions described in the paper [12]. The average score and standard deviation for each item and each school year among the students were expressed in a previous paper [3].

### 2.2. The Items in SMQ-II and R-SPQ-2F are Listed in Supplementary Material 1

Statistical analysis: The structural equation modeling (SEM), including multi-group analysis, was performed using AMOS version 24 (IBM Japan, Tokyo, Japan). The goodness of fit (GOF) of the model with the data was examined using several GOF statistics, such as Chi-square (minimum discrepancy/degree of freedom: CMIN/DF), goodness-of-fit index (GFI), root mean square error of approximation (RMSEA), comparative fit index (CFI) and Akaike information criteria (AIC) [13]. A non-significant Chi-square test indicates that the data fit the model. The GFI measures the relative amount of variance and covariance accounted for by the model, which varies between 0 (no fit) and 1 (perfect fit). The RMSEA is an average error in predicted values and actual values of all correlations between the pairs of variables in the model. A smaller RMSEA (usually less than 0.05) indicates a better fit of the model for the data. The CFI indicates how much this model fits better than a model with no relationships between any of the variables. A greater CFI (usually > 0.9) indicates a model with a better fit. AIC is an estimator of the relative quality of statistical models for a given set of data and is used to compare models. 

The effect sizes were calculated using G*Power, version 3.1.9.2. (The Faculty of Mathematics and Natural Sciences, Heinrich Heine University Düsseldorf) [14]. 

The ethics committee of the Faculty of Pharmaceutical Sciences at Josai International University approved the survey (ID of the approval: 45).

## 3. Results

### Causal Relationship of Learning Motivation Profiles and Learning Process

**Figure 1:**
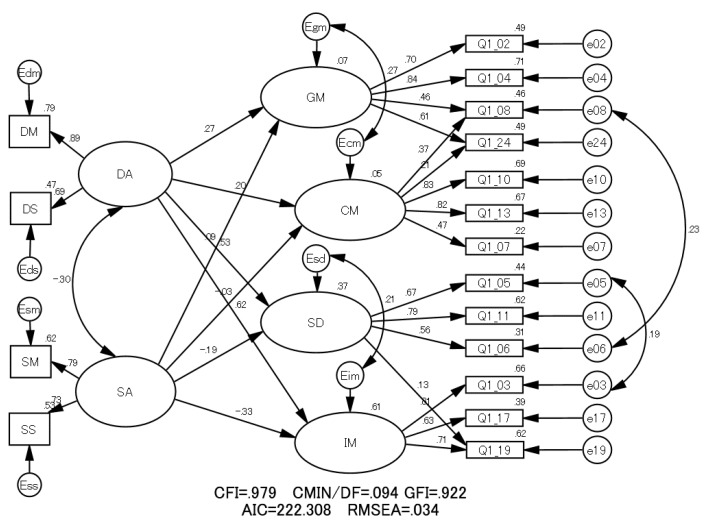
The structure of Model 1 with standardized estimators among variables and goodness-of-fit (GOF) statistics. In this figure, DA: deep approach, SA: surface approach, DM: deep motive; DS: deep strategy, SM: surface motive, SS: surface strategy, GM: grade motivation, CM: career motivation, SD: self-determination and IM: intrinsic motivation.

**Figure 2:**
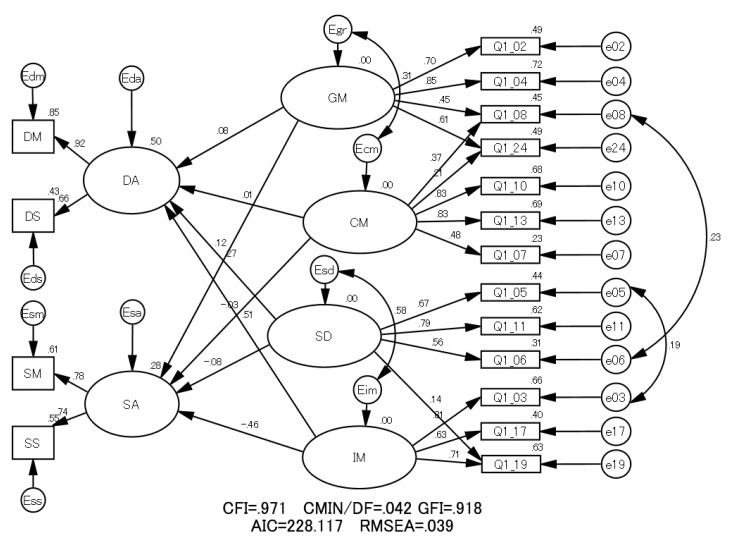
The structure of Model 2 with standardized estimators among variables and GOF statistics. In this figure, DA: deep approach, SA: surface approach, DM: deep motive; DS: deep strategy, SM: surface motive, SS: surface strategy, GM: grade motivation, CM: career motivation, SD: self-determination and IM: intrinsic motivation.

The authors postulated two possible models related to the ideas that the learning process affects students’ learning motivation profiles (Model 1) or that the learning motivation profile affects students’ learning process (Model 2). The path diagrams of Model 1 and Model 2 are shown in Figure 1 and Figure 2 with results of SEM analysis. The left and right parts of these figures depict the learning process and learning motivation profile, respectively. 

In Figure 1 and Figure 2, the single-headed and double-headed arrows indicate causal relationships and correlations between variables, respectively. In Model 1, the arrows were drawn from the latent variables of learning process (DA and SA) to those of the learning motivation profile (GM, CM, SD and IM). On the other hand, in Model 2, the arrows were drawn from the latent variables of learning motivation profile to those of learning process. Models 1 and 2 are founded on the ideas that the learning process affects students’ learning motivation profiles and that the learning motivation profile affects students’ learning process, respectively.

To establish a model with a better fit, some correlations were added to the original models [2,3].

Both Model 1 and Model 2 satisfied most GOF statistics in terms of the conventional criteria (*p*-values of the Chi-square test, CMIN/DF: >0.05; CFI: >0.90; GOF index: (GFI): >0.90; RMSEA: <0.05) [15]. 

Comparing the GOF statistics, Model 1 was found to be a better fit for the data than Model 2 because CFI and GFI were larger and RMSEA and AIC were smaller in comparison with Model 2 [13,15]. Thus, the authors adopted Model 1 as the structure illustrating the causal relationship between the learning process and learning motivation profile. 

Table 1 shows the estimated regression weights from the learning process to the learning motivation profile in Model 1. All estimated regression weights of the paths from DA to learning motivation factors were all positive and statistically significant (less than 0.05). In contrast, the regression weights from SA to the components of learning motivation were not significant or negative. These results indicate that DA helped to increase learning motivation, while SA had little influence or a slightly negative influence on learning motivation. 

#### Change in Learning Structure by School Year

Based on Model 1, the authors assessed the change in relationship between the learning process and learning motivation profile by school year using the multi-group analysis. In multi-group analysis, several constrained models can be established. The smallest AIC was found in the constrained model, which had equalized measurement weights, measurement intercepts and structural weights among the school years. Therefore, this model was selected to be the final model. The influence of learning process on the learning motivation profile was evaluated from the change in estimated means of DA and SA by school year. 

Table 2 illustrates the estimated means of DA and SA components in terms of the learning motivation profile in 5th- and 6th-year students compared with those in 4th-year students. Furthermore, the effect size of the difference was calculated in the form of Cohen’s *d* [16]. The estimated mean of DA was found to increase with a higher school year. A significant difference of the estimated mean in DA between 4th- and 6th-year students was found to be 1.264 (*p* = 0.006), with a medium effect size of *d* = 0.471 [16]. The change in the estimated mean of SA was not significant.

## 4. Discussion

To establish the model of Japanese pharmacy students’ learning structure, the authors gathered the sample data using questionnaires to assess the learning motivation profiles and learning process of 4th-year to 6th-year students. This is because some 1st- to 3rd-year pharmacy students change their major by their 4th year. The teaching curriculum from the 4th year onward is more clinically oriented. The authors are interested in understanding how clinical experience can change the learning structure because clinical experience for pharmacy students would be important in developing a pharmacist who pursues life-long learning. 

It is known that the students’ learning process and learning motivation are related to each other. The DA has been reported to correlate with students’ intrinsic interest and their ability to maximize meaning [7,17]. Kusurker et al. reported that deep learning was strongly associated with the interest-motivated profile, which is similar to the IM among medical students [8]. Although DA was known to be associated with learning motivation, a causal relation between these two variables would be usually unclear. In this report, we revealed that the learning process influenced the learning motivation in Japanese pharmacy students. 

From the multi-group analysis, the estimated mean of DA increased statistically in their 6th year and the effect size was almost medium. An increase in the estimated mean of DA indicated that the average effect of DA on variables become larger. This would show that the pharmacy students adopted deep learning processes in their 6th year. 

The educational environment has been reported to enhance SD motivation in health professional education [9,18]. A learning environment focusing on patient-centered education seems to improve both deep and active learning as well as IM [17]. There are also reports that the students adopted deep learning during experiential education [19,20]. These findings suggest that clinical clerkship can help to develop deep learning process and improve the learning motivation of pharmacy students. As the learning process influences their learning motivation profiles, the DA developed by clinical clerkships is considered to affect their learning motivation. 

If pharmacy students adopt deep learning, they can learn the meaning behind the pharmaceutical knowledge that they have learned already in pharmacy schools and will be more engaged in learning. Thus, they are more motivated to learn in the long term [4]. Our model expressed that the DA developed by clinical clerkships increase the dominantly learning motivation as SD and IM.

Overall, a clinical clerkship can enhance students’ deep learning profile and increase their learning motivation. Pharmacy schools and preceptors should know that they would play a new role in enhancing the learning motivation accompanied with developing deep learning skills of pharmacy students. If we can set the appropriate goals for deep learning to be achieved in clinical clerkship, we can educate highly motivated pharmacy students together with pharmacy schools and preceptors. 

## 5. Conclusions

Our model revealed that the deep learning process of pharmacy students was developed during clinical clerkship and the deep learning can enhance their learning motivation profiles. The development of deep learning skills would be a new aim of preceptors during clinical clerkships. 

Regarding limitations, the present study was only conducted at one Japanese pharmacy school. Future research using data from other international pharmacy schools might be necessary to develop a more sophisticated model. Pharmacy students have “profession-oriented” learning motivation since they want to be healthcare professionals. Further surveys should be carried out using a questionnaire that includes the “profession-oriented” factor. 

## Figures and Tables

**Table 1:** Standardized estimated regression weights from the learning process (DA and SA) to learning motivation factors (GM, CM, SD and IM) in Model 1.

Path	Standardized Estimate	Estimate	Standard Error	*p*-Value
DA to GM	0.270	0.078	0.030	0.009
DA to CM	0.204	0.062	0.031	0.045
DA to SD	0.530	0.148	0.031	<0.001
DA to IM	0.618	0.168	0.028	<0.001
SA to GM	0.090	0.028	0.033	0.397
SA to CM	–0.035	−0.012	0.035	0.742
SA to SD	–0.188	−0.057	0.031	0.069
SA to IM	–0.326	−0.096	0.029	<0.001

*p*-values were obtained from estimates and their standard errors by the *z*-test. In this table, DA: deep approach, SA: surface approach, GM: grade motivation, CM: career motivation, SD: self-determination and IM: intrinsic motivation.

**Table 2:** Estimated means for the components of DA and SA.

Valuable	Pairs	Estimated Mean	Standard Error	*p*-Value	Effect Size (Cohen’s *d*)
DA	5th-year and 4th-year students	0.560	0.645	0.385	0.172
	6th-year and 4th-year students	1.264	0.464	0.006	0.471
SA	5th-year and 4th-year students	0.557	0.587	0.343	0.209
	6th-year and 4th-year students	–0.241	0.478	0.614	0.102

*p*-values were obtained from estimates and their standard errors by the *z*-test. In this table, DA: deep approach and SA: surface approach.

## Supplementary Materials

Supplementary File 1
