# Supplementary material for: An Analysis of the Relationship between the Learning Process and Learning Motivation Profiles of Japanese Pharmacy Students Using Structural Equation Modeling
Source: Pharmacy (Basel). 2018 Apr 23;6(2):35. doi: 10.3390/pharmacy6020035 (PMC6024982; doi:10.3390/pharmacy6020035)
Supplement: Supplementary File 1 [file pharmacy-06-00035-s001.zip › pharmacy-279126-SI.pdf]

pharmacy

Questionnaires of SMQ-II and SPQ-2

**1. Questionnaire of SMQ-II**

Intrinsic Motivation component

Q1_01. The science I learn is relevant to my life.

Q1_03. Learning science is interesting.

Q1_12. Learning science makes my life more meaningful.

Q1_17. I am curious about discoveries in science.

Q1_19. I enjoy learning science.

Self-Efficacy component

Q1_09. I am confident I will do well on science tests.

Q1_14. I am confident I will do well on science labs and projects.

Q1_15. I believe I can master science knowledge and skills.

Q1_18. I believe I can earn a grade of "A" in science.

Q1_21. I am sure I can understand science.

Self-Determination component

Q1_05. I put enough effort into learning science.

Q1_06. I use strategies to learn science well.

Q1_11. I spend a lot of time learning science.

Q1_16. I prepare well for science tests and labs.

Q1_22. I study hard to learn science.

Grade Motivation component

Q1_02. I like to do better than other students on science tests.

Q1_04. Getting a good science grade is important to me.

Q1_08. It is important that I get an "A" in science.

Q1_20. I think about the grade I will get in science.

Q1_24. Scoring high on science tests and labs matters to me.

Career Motivation component

Q1_07. Learning science will help me get a good job.

Q1_10. Knowing science will give me a career advantage.

Q1_13. Understanding science will benefit me in my career.

Q1_23. My career will involve science.

Q1_25. I will use science problem-solving skills in my career.

**2. Questionnaire of SPQ-2**

Q2_01. I find that at times studying gives me a feeling of deep personal satisfaction.

Q2_02. I find that I have to do enough work on a topic so that I can form my own conclusions before I am satisfied.

Q2_03. My aim is to pass the course while doing as little work as possible.

Q2_04. I only study seriously what's given out in class or in the course outlines.

Q2_05. I feel that virtually any topic can be highly interesting once I get into it.
Q2_06. I find most new topics interesting and often spend extra time trying to obtain more information about them.
Q2_07. I do not find my course very interesting so I keep my work to the minimum.
Q2_08. I learn some things by rote, going over and over them until I know them by heart even if I do not understand them.
Q2_09. I find that studying academic topics can at times be as exciting as a good novel or movie.
Q2_10. I test myself on important topics until I understand them completely.
Q2_11. I find I can get by in most assessments by memorising key sections rather than trying to understand them.
Q2_12. I generally restrict my study to what is specifically set as I think it is unnecessary to do anything extra.
Q2_13. I work hard at my studies because I find the material interesting.
Q2_14. I spend a lot of my free time finding out more about interesting topics which have been discussed in different classes.
Q2_15. I find it is not helpful to study topics in depth. It confuses and wastes time, when all you need is a passing acquaintance with topics.
Q2_16. I believe that lecturers shouldn't expect students to spend significant amounts of time studying material everyone knows won't be examined.
Q2_17. I come to most classes with questions in mind that I want answering.
Q2_18. I make a point of looking at most of the suggested readings that go with the lectures.
Q2_19. I see no point in learning material which is not likely to be in the examination.
Q2_20. I find the best way to pass examinations is to try to remember answers to likely questions.

Deep motive (DM): Q2_01+Q2_05+Q2_09+Q2_13+Q2_17
Deep strategy (DS): Q2_02+Q2_06+Q2_10+Q2_14+Q2_18
Surface motive (SM): Q2_03+Q2_07+Q2_11+Q2_15+Q2_19
Surface strategy (SS): Q2_04+Q2_08+Q2_12+Q2_16+Q2_20
Deep approach (DA): DM+DS
Surface approach (SA): SM+SS
